# A Comparative ^68^Ga-Citrate and ^68^Ga-Chloride PET/CT Imaging of* Staphylococcus aureus* Osteomyelitis in the Rat Tibia

**DOI:** 10.1155/2018/9892604

**Published:** 2018-02-25

**Authors:** Petteri Lankinen, Tommi Noponen, Anu Autio, Pauliina Luoto, Janek Frantzèn, Eliisa Löyttyniemi, Antti J. Hakanen, Hannu T. Aro, Anne Roivainen

**Affiliations:** ^1^Department of Orthopaedics and Traumatology, Turku University Hospital, Turku, Finland; ^2^Department of Clinical Physiology and Nuclear Medicine, Turku University Hospital and University of Turku, Turku, Finland; ^3^Turku PET Centre, University of Turku, Turku, Finland; ^4^Division of Clinical Neurosciences, Department of Neurosurgery, Turku University Hospital, Turku, Finland; ^5^Department of Biostatistics, University of Turku, Turku, Finland; ^6^Department of Clinical Microbiology, Turku University Hospital and Medical Microbiology and Immunology, University of Turku, Turku, Finland; ^7^Orthopaedic Research Unit, Department of Orthopaedic Surgery and Traumatology, University of Turku, Turku, Finland; ^8^Turku PET Centre, Turku University Hospital, Turku, Finland; ^9^Turku Center for Disease Modeling, University of Turku, Turku, Finland

## Abstract

There may be some differences in the* in vivo* behavior of ^68^Ga-chloride and ^68^Ga-citrate leading to different accumulation profiles. This study compared ^68^Ga-citrate and ^68^Ga-chloride PET/CT imaging under standardized experimental models.* Methods.* Diffuse* Staphylococcus aureus* tibial osteomyelitis and uncomplicated bone healing rat models were used (*n* = 32). Two weeks after surgery, PET/CT imaging was performed on consecutive days using ^68^Ga-citrate or ^68^Ga-chloride, and tissue accumulation was confirmed by* ex vivo* analysis. In addition, peripheral quantitative computed tomography and conventional radiography were performed. Osteomyelitis was verified by microbiological analysis and specimens were also processed for histomorphometry.* Results.* In PET/CT imaging, the SUV_max_ of ^68^Ga-chloride and ^68^Ga-citrate in the osteomyelitic tibias (3.6 ± 1.4 and 4.7 ± 1.5, resp.) were significantly higher (*P* = 0.0019 and *P* = 0.0020, resp.) than in the uncomplicated bone healing (2.7 ± 0.44 and 2.5 ± 0.49, resp.). In osteomyelitic tibias, the SUV_max_ of ^68^Ga-citrate was significantly higher than the uptake of ^68^Ga-chloride (*P* = 0.0017). In animals with uncomplicated bone healing, no difference in the SUV_max_ of ^68^Ga-chloride or ^68^Ga-citrate was seen in the operated tibias.* Conclusions.* This study further corroborates the use of ^68^Ga-citrate for PET imaging of osteomyelitis.

## 1. Introduction

Deep bone infections are one of the most challenging conditions to treat in orthopedics and trauma surgery. By nature, these infections are highly challenging for diagnostic imaging, especially when bone structures have been altered by trauma, surgery, or a previous pathological condition. Standard diagnostic tools such as conventional radiographs, magnetic resonance imaging (MRI), computed tomography (CT), and conventional nuclear medicine methods are known to have major limitations in the early diagnosis of deep bone infections [1–5]. Positron emission tomography (PET) imaging with 2-deoxy-2-[^18^F]-fluoro-*D*-glucose (^18^F-FDG) has been shown to have a high diagnostic accuracy for confirming or excluding the diagnosis of chronic osteomyelitis [3, 6, 7]. In the clinical setting, the use of ^18^F-FDG-PET for differential diagnosis involves a risk of possible false positive findings due to the early bone healing process, which involves an inflammatory phase. This phase represents a highly activated state of cell metabolism and glucose consumption [8] and can thus possibly mimic a similar ^18^F-FDG-PET tracer uptake pattern to that occurring during infection. In an experimental study using a rabbit osteomyelitis model, bone infection could be distinguished from bone healing by means of ^18^F-FDG PET 3 weeks after surgery [9]. In a previous rat study, we reported elevated uptake of ^18^F-FDG in healing bone but a significantly lower uptake of ^68^Ga-chloride in tibias with uncomplicated healing defects, whereas no statistical difference was seen between the tracers in osteomyelitic tibias [10]. Thus, in patients with postsurgical and posttraumatic bone healing, ^68^gallium could be more promising than ^18^F-FDG in the discrimination of bacterial infection from the unspecific uptake caused by the physiological inflammatory processes of normal bone healing.

^67^Ga-citrate has been used in scintigraphy for the evaluation of infectious processes for several decades. The accumulation of gallium in inflammatory or infectious sites is partly due to the increased capillary permeability associated with inflammatory reactions; gallium exits the vascular network and is trapped in the extravascular compartment [11]. As an iron analogue, it binds to circulating transferrin and, via transferrin receptors, accesses cells and evolves to a highly stable state [12]. As a tracer, gallium is able to bind to bacterial siderophores and activated lactoferrin in neutrophils [13], with an uptake by macrophages also having been demonstrated [14–16]. In addition, a direct bacterial uptake pattern has been reported [17]. In general, different radionuclides such as ^68^Ga (positron emitter used for PET) and ^67^Ga (gamma emitter used for single-photon emission computed tomography (SPECT)) vary only according to their physical properties, with their chemical and physiological behavior being comparable. However, there may be some differences between ^68^Ga-chloride and ^68^Ga-citrate in regard to their* in vivo* behavior.

The purpose of this study was to compare ^68^Ga-citrate and ^68^Ga-chloride PET/CT imaging under standardized experimental models of uncomplicated bone healing and* Staphylococcus aureus* osteomyelitis. In addition,* ex vivo* measurements of tissue radioactivity concentration in normal bone healing and osteomyelitic tissues were performed to verify the uptake and biodistribution of ^68^Ga-citrate.

## 2. Materials and Methods

### 2.1. Animals

Thirty-two skeletally mature male Sprague–Dawley rats (Harlan, Horst, The Netherlands) with a mean weight of 412.6 ± 64.2 g were used in these experiments. The rats were allowed to acclimatize to their new environment before surgery. All animal experiments were approved by the National Animal Experiment Board in Finland and the Regional State Administrative Agency for Southern Finland and were conducted in accordance with the European Union directive.

### 2.2. Experimental Design

**Figure 1:**
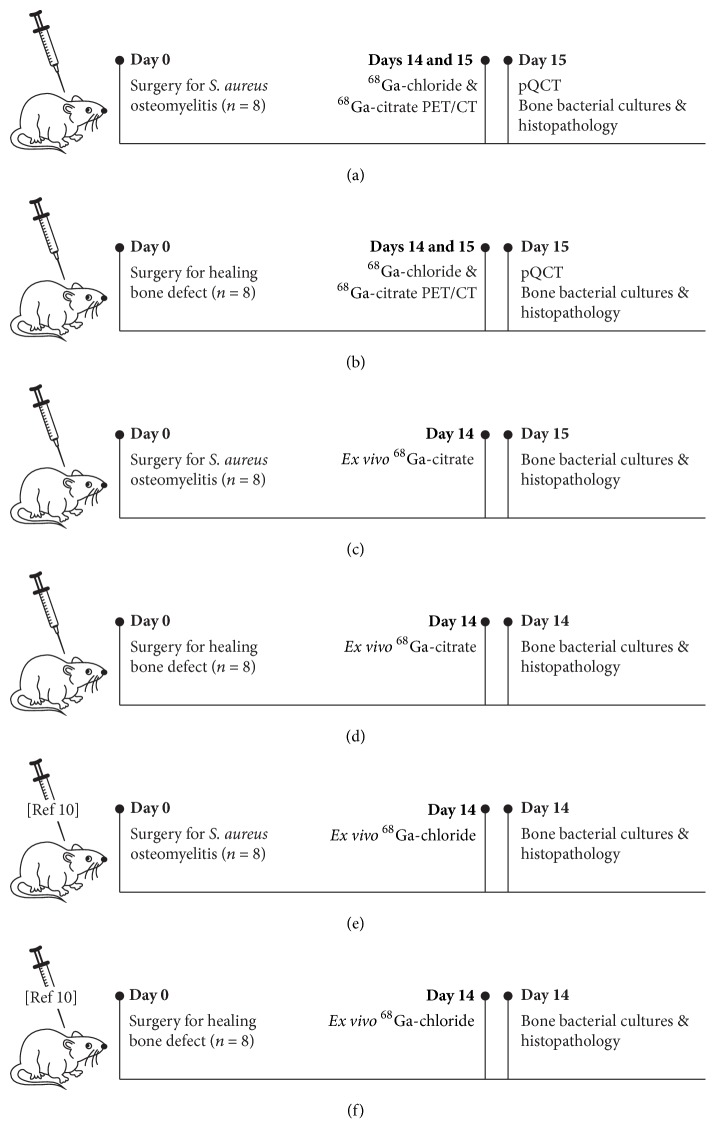
Schematic illustration of experimental design and timing of performed analysis methods.

In each animal, the left tibia was operated on and the right contralateral tibia served as an intact control. For the comparative ^68^Ga-citrate and ^68^Ga-chloride PET/CT imaging, animals with induced osteomyelitis (*n* = 8) and animals with normal bone healing (*n* = 8) were imaged with both tracers on consecutive days (Figure 1). Bone structural changes caused by infection were evaluated by peripheral quantitative computed tomography (pQCT), which was performed after the PET/CT imaging. The operated tibias were harvested and samples were retrieved for quantitative microbiological analyses and semiquantitative histopathologic analyses. For the* ex vivo* measurements of tissue radioactivity concentration, the accumulation of ^68^Ga-citrate was studied in animals with induced osteomyelitis (*n* = 8) and animals with normal bone healing (*n* = 8). All studies were performed 2 weeks after surgery, that is, after induction of osteomyelitis or creation of a healing bone defect.

### 2.3. Induction of Infection

The diffuse rat osteomyelitis model (stage IVA in the Cierny-Mader classification; osteomyelitis secondary to a contiguous focus of infection in the Waldvogel classification) was adopted for this study [10, 18–20]. The rats were anaesthetized with a mixture of fentanyl, fluanisone (Hypnorm, Janssen Pharmaceutica, Beerse, Belgium), and midazolam (Midazolam Hameln 5 mg/ml, Hameln Pharmaceuticals GmbH, Hameln, Germany). The left hind leg was shaved, disinfected, and covered with sterile sheets. Using sterile surgical conditions, a small cortical bone defect (diameter 1.0 mm) was created into the proximal medial metaphysis of the right tibia using a high speed dental drill. Local bone marrow was removed with saline lavage. As described earlier [10, 18], osteomyelitis was induced by injecting into the medullary cavity a volume of 0.05 ml of 5% wt/vol sodium morrhuate (Scleromate, Glenwood, Englewood, NJ, USA), which was immediately followed by a 0.05 ml volume of the bacterial inoculum (3 × 10^8^ colony-forming unit [CFU]/ml of* S. aureus*). The drilling hole was sealed with bone wax (Braun, Aesculap AG & Co., Tuttlingen, Germany) to prevent bacterial leakage and provide a foreign body for infection [20]. Finally, the skin wound was cleaned with a 40 ml sterile saline lavage without antibiotics and closed in layers. In control animals, a cortical defect of equal size was drilled, but the sodium morrhuate, bacterial suspension, and bone wax were not used. Before wound closure, the surgical field was lavaged with 40 ml sterile saline containing 150 mg cefuroxime sodium (Zinacef, GlaxoSmithKline, Verona, Italy). Bacitracin and neomycin sulfate powder (Bacibact, Orion Oyj, Espoo, Finland) were applied to the sutured skin wound and it was covered with an aerosol-based plastic film (HansaPlast, Beiersdorf AG, Hamburg, Germany). Anaesthesia was reversed by a subcutaneous injection of naloxone (Narcanti, Du Pont Pharmaceuticals Ltd., Letchworth, UK). After surgery, the animals were closely monitored and standard postoperative pain medication was given for the first 3 postoperative days (0.5 mg/kg of buprenorphine subcutaneously every 12 hours, Temgesic® 0.3 mg/ml, Schering-Plough, Brussels, Belgium). The animals were housed individually for 2 days, after which they were returned to their normal housing in groups of two. All the animals had an uneventful postoperative recovery, and their activity was not limited within the individual cages.

### 2.4. ^68^Ga-Citrate and ^68^Ga-Chloride PET/CT

Comparative ^68^Ga-citrate and ^68^Ga-chloride PET/CT were performed 2 weeks after the surgery in which either osteomyelitis was induced or a cortical bone defect was created (Figure 1).

To prepare the ^68^Ga-chloride injection, ^68^gallium from ^68^Ge/^68^Ga generator (Eckert & Ziegler, Valencia, CA, USA) was eluted with 0.1 M hydrochloric acid and neutralized with 1 M sodium hydroxide before use. ^68^Ga-citrate was prepared by mixing the ^68^Ga-eluate with sodium citrate as previously described [21]. Radiochemical purity was evaluated by the instant thin layer chromatography-silica-gel technique using methanol/acetic acid (9 : 1) as a mobile phase.

PET/CT imaging was performed with a Discovery VCT (General Electric Medical Systems, Milwaukee, WI, USA) operating in 3-dimensional mode. This is a combined 64-slice CT and PET scanner with 24 rings of bismuth germanate detectors; it acquires 47 imaging planes with an axial field-of-view of 15.7 cm. The transaxial crystal size of the PET scanner is 4.7 mm, and the spatial resolution in 3D mode is 5.12 mm in full width at half maximum with a 1 cm offset from the center of the field-of-view [22]. In a pilot study, two rats in the osteomyelitis group underwent 180 min dynamic scanning starting immediately after an i.v. injection of ^68^Ga-citrate. The protocols for PET imaging were designed according to the observed patterns of tracer accumulation. Dynamic PET imaging consisting of 4 × 5 min frames was started 120 minutes after the injection of ^68^Ga-citrate and 90 minutes after the injection of ^68^Ga-chloride. Dynamic PET imaging was performed to avoid the potential effects of animal movement during the scanning period. CT was performed before PET, using the following technical parameters: helical scan mode, helical slice thickness of 3.75 mm, detector coverage of 20 mm, pitch factor of 0.531 : 1, voltage of 100 kVp, current of 80 mA, rotation time of 1 s, “large body” scan field-of-view, and display field-of-view 50. PET images were reconstructed using an ordered subsets expectation maximization algorithm and the CT images were reconstructed using a CT attenuation correction (CTAC) and bone kernels. The CT data were used for attenuation correction (CTAC images) and anatomical reference (bone images) when fused with the PET images.

The animals fasted for 4 h prior to tracer injection and were sedated for PET/CT imaging as in the surgical procedure. On average, 29.1 ± 1.8 MBq of ^68^Ga-citrate (mean ± standard deviation [SD]) and 28.8 ± 2.4 MBq of ^68^Ga-chloride were injected into the tail vein of the animal in a volume of 0.5–1.0 ml. Quantitative analysis of tracer uptake was performed for a standardized circular region of interest (ROI diameter, 3.0 mm) in the operated left tibia and the corresponding region in the contralateral intact right tibia. The levels of ^68^Ga-citrate and ^68^Ga-chloride accumulation were reported as the maximum standardized uptake value (SUV_max_). The SUV_max_ was calculated as the maximum radioactivity concentration within the ROI divided by the relative injected radioactivity dose expressed per kg of body weight. In addition, SUV ratios, that is, operated bone-to-intact bone, were calculated.

### 2.5. pQCT

Each animal underwent pQCT scanning following the PET imaging. Under fentanyl-fluanisone sedation, the operated limbs were placed in a holder for standard positioning. Imaging was performed using a Stratec XCT Research M pQCT device with software version 5.20 (Norland Stratec Medizintechnik GmbH, Birkenfeld, Germany). After an initial scout view for positioning, the proximal tibias were imaged with six consecutive cross-sectional images using a slice distance of 0.75 mm. A voxel size of 0.07 × 0.07 × 0.50 mm was used. The pQCT images were analysed for the presence of osteomyelitic destruction and reactive new bone formation.

After pQCT imaging, the animals were killed with an intravenous administration of sodium pentobarbital.

### 2.6. Microbiological Analyses

The presence of infection was confirmed with bacterial cultures at the time of killing. Using sterile techniques, the bone defect area was exposed and swab cultures were taken from subfascial soft tissues. The proximal tibia was aseptically cross-sectioned using a high speed circular saw to obtain three (proximal, middle, and distal) bone specimens from the site of bone infection. The proximal and middle segments were used for histological analysis and the distal bone segment was used for quantitative bacterial culture.

All swab specimens were cultured for 18–20 hours at 35°C on blood agar plates. After snap-freezing in liquid nitrogen and homogenization with a mortar and pestle, the distal bone segment was vortexed in saline for 5 min, and ten serial 10-fold dilutions were performed to determine the CFU of* S. aureus* per gram of bone. The dilutions were cultured for 18–20 hours at 35°C on blood agar plates. The aseptically harvested bone cement blocks were cultured on blood agar and immediately placed in BBL™ Brain Heart Infusion broth (Becton, Dickinson and Company, Sparks, MD, USA) and incubated for up to 5 days at 35°C. The turbidity of broth samples was observed every day, and positive cultures (i.e., opaque tubes) were plated onto blood agar plates and incubated for 18–20 hours at 35°C. Negative broth samples (i.e., clear tubes) were similarly cultured after 2 and 5 days of incubation.

The isolated pathogens were identified on the basis of their morphology and with the Slidex® Staph Plus latex agglutination test (bioMérieux, Marcy l'Etoile, France) [23].* S. aureus* (American Type Culture Collection [ATCC] strain 29213) was used as the positive control and* Enterococcus faecalis* (ATCC strain 29212) was used as the negative control.

### 2.7. Semiquantitative Histopathologic Analysis

The proximal and middle bone specimens were processed for histology. The proximal specimen was fixed in 70% ethanol, embedded in isobornyl methacrylate (Technovit 1200 VLC, Kulzer, Germany), and stained with a modified van Gieson method. The middle bone segment was decalcified, embedded in paraffin, and stained with hematoxylin and eosin. Histological changes in the periosteum, cortical bone, and medullary canal were classified according to the histopathological scoring system presented by Petty and coworkers [10, 18, 24, 25]. Two observers classified the histological sections, with the results presented being their mutual agreement of the interpretation.

### 2.8. *Ex Vivo* Measurement of ^68^Ga-Citrate Accumulation

As a separate substudy, the accumulation of ^68^Ga-citrate at 2 weeks after operation was studied* ex vivo* in animals with induced osteomyelitis (*n* = 8) and control animals with a healing bone defect (*n* = 8) (Figure 1). The animals fasted for 4 h prior to tracer injection. Under sedation obtained by a subcutaneous injection of midazolam, fluanisone, and fentanyl citrate, a dose of 33.0 ± 3.2 MBq of ^68^Ga-citrate was injected via the tail vein of the animal in a volume of 0.5–1.0 ml. After tracer accumulation (90 min for ^68^Ga-citrate), a blood sample was obtained via intracardiac puncture, and the animals were sacrificed by intracardiac administration of sodium pentobarbital (Mebunat, Orion, Espoo, Finland). Quantitative bacterial culture was performed on bone tissue specimens removed from the distal tibia to confirm the presence of osteomyelitis. In addition to tissue specimens excised from calf muscles, a 15 mm long segment of the operated proximal tibia (including the site of the bone defect) and a corresponding segment of the contralateral tibia were resected for analysis of tracer accumulation. The radioactivity of blood, muscle, and bone specimens was measured with a gamma counter (1480 Wizard 3′′; PerkinElmer/Wallac, Turku, Finland) cross-calibrated with a dose calibrator (VDC-202, Veenstra Instruments, Joure, The Netherlands). The radioactivity concentration was expressed as SUV [(tissue radioactivity/tissue weight)/(total given radioactivity/rat body weight)], and the SUV ratios (operated bone-to-muscle, operated bone-to-blood and operated bone-to-intact bone) were calculated.

We used previously published results of* ex vivo*^68^Ga-chloride accumulation that utilized the same study protocol and identical animal model [10] to make comparisons with the accumulation of ^68^Ga-citrate (Figure 1).

### 2.9. Statistical Analysis

Data are expressed as mean ± SD. Histological osteomyelitic changes (periosteal reaction, cortical bone, and medullary canal) were compared between the groups with a Wilcoxon rank sum test. The comparisons of SUV ratios between ^68^Ga-citrate and ^68^Ga-chloride, and between the osteomyelitis and bone healing groups, were performed with a hierarchical linear mixed model for repeated measures, including one within-factor (tracer) and one between-factor analysis (group). The model also included interaction between the factors, which indicated whether a significant mean difference between the groups was different between the two different tracers. Pairwise comparisons between tracers for specific study questions were programmed into the model. SUV_max_ was analysed separately for both tracers, using similar methods.

All tests performed were two-sided, with a significance level set at 0.05. The analyses were performed using SAS version 9.3 (SAS Institute Inc., Cary, NC, USA).

## 3. Results

### 3.1. Confirmation of Staphylococcal Infection and Histological Appearance of Osteomyelitis

The inoculated pathogen was cultured from the homogenized bone specimens in all animals with induced osteomyelitis. In 14 out of 16 osteomyelitic animals (87.5%), the swab cultures taken from subfascial soft tissues were positive for the inoculated* S. aureus*, indicating the extension of infection outside the bone. None of the animals had an infected draining sinus. No bacteria could be cultured from the homogenized bone specimens retrieved from the control animals. Similarly, all swab cultures from the soft tissues of control animals were negative.

**Figure 2:**
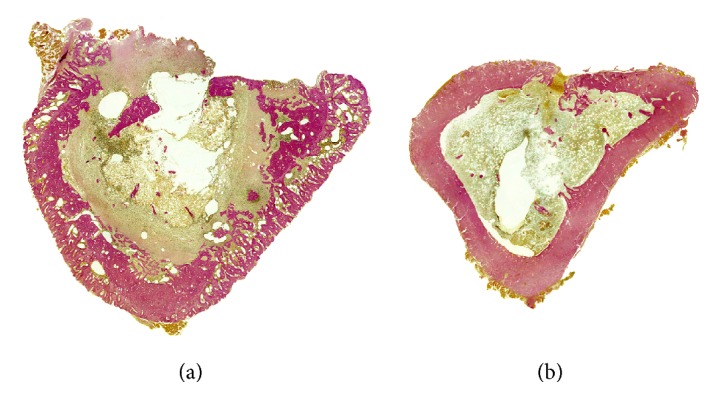
Histological sections of osteomyelitic (a) and control (b) rat tibias at 2 weeks after surgery. The osteomyelitic changes were characterised by a wide circumferential periosteal reaction, focally enlarged haversian canals filled with fragmented polymorphonuclear leukocytes and occasional microabscesses, and major infiltration of the bone marrow by polymorphonuclear leukocytes. In some cases, a devitalized bone fragment was seen in the unhealed cortical window. In the control animals, periosteal reaction was minimal and there was modest endosteal new bone close to the cortical defect, indicating healing of the cortical defect. Modified van Gieson stain at ×10 magnification.

The* S. aureus* group of animals showed histologically severe osteomyelitis in all cases (histologic score 2.5 ± 0.4; Figure 2). Histological appearance was characterised by a nearly circumferential periosteal reaction, reactive new bone formation, occasional sequester formation, and drastic infiltration of polymorphonuclear leukocytes with occasional microabscesses. The control group with healing cortical-defects showed healing of the defect by endosteal new bone formation, with no signs of infection (score 0.2 ± 0.3; Figure 2). Histological appearance was characterised by closure of the cortical defect with only a limited number of inflammatory cell infiltrations. There was a significant difference in the mean histological score between the two groups (*P* = 0.0065).

### 3.2. ^68^Ga-Citrate and ^68^Ga-Chloride PET/CT Imaging

**Figure 3:**
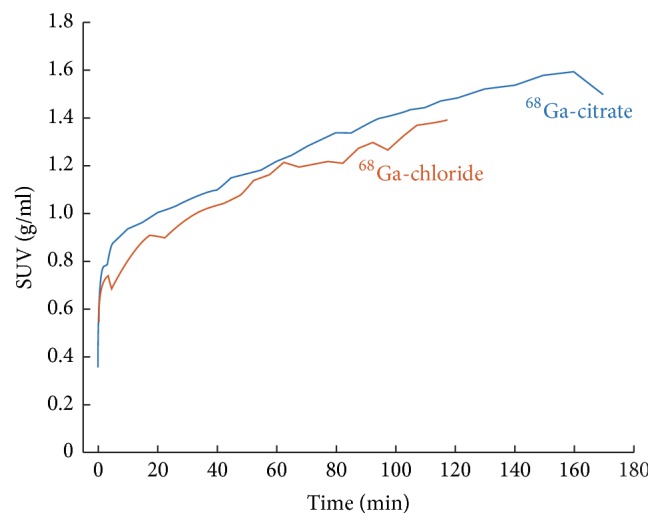
Time-activity curves for ^68^Ga-citrate and ^68^Ga-chloride accumulation at the site of induced osteomyelitis in rat tibia as determined by* in vivo* PET/CT imaging. The line represents the mean value of two animals. The radioactivity concentration, expressed in SUV, has been decay-corrected to the time of injection.

**Figure 4:**
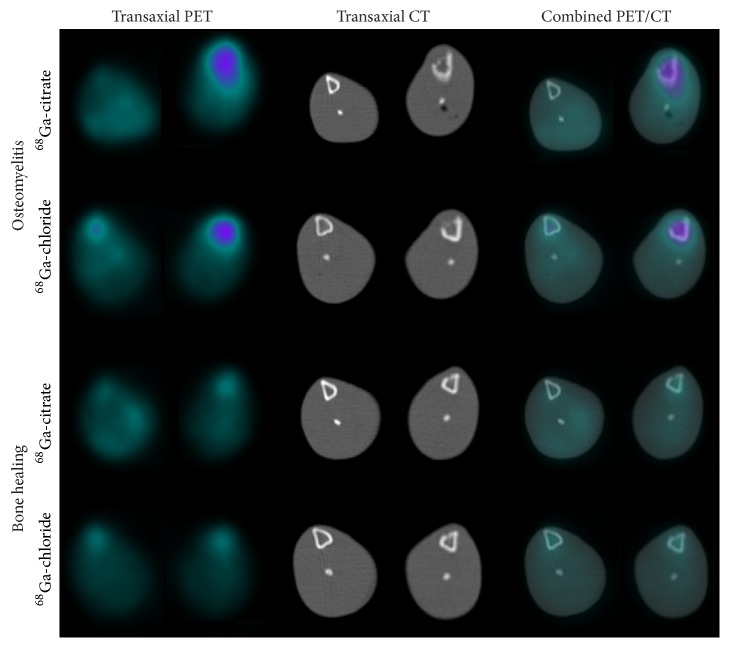
Representative transaxial PET, CT, and combined PET/CT images of ^68^Ga-citrate and ^68^Ga-chloride accumulation at the site of induced osteomyelitis and healing bone-defects at 2 weeks after surgery. In each animal, the left tibia (on the right) underwent surgery for induction of infection or the creation of a surgical defect to represent uncomplicated bone healing, with the contralateral intact bone (on the left) serving as the control.

On the basis of the dynamic imaging, the accumulation kinetics of ^68^Ga-chloride and ^68^Ga-citrate at the site of infection was found rather stable at 90–120 min postinjection (Figure 3). PET/CT imaging demonstrated intense accumulation of both ^68^Ga-chloride and ^68^Ga-citrate in osteomyelitic tibias in comparison with the contralateral intact bone or animals with uncomplicated bone healing (Figure 4).

**Figure 5:**
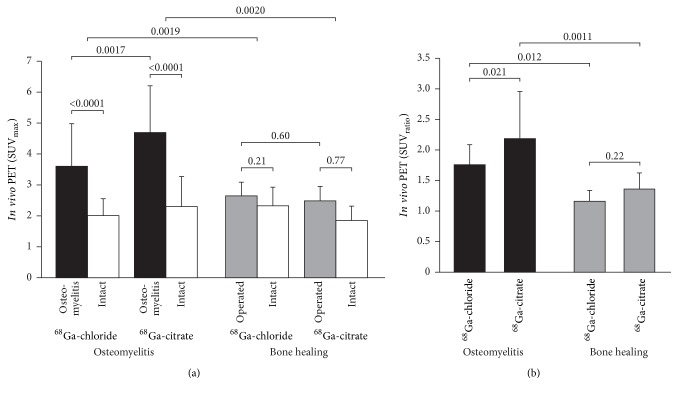
Quantification of ^68^Ga-citrate and ^68^Ga-chloride PET/CT imaging at 2 weeks after surgery. Bar graphs represent mean SUV_max_ values (±SD) (a) and SUV_max_ ratios, that is, operated bone-to-intact bone (b) (*n* = 8).

The SUV_max_ of ^68^Ga-chloride and ^68^Ga-citrate in the osteomyelitic tibias (3.6 ± 1.4 and 4.7 ± 1.5, respectively, *P* = 0.0017 between them) were significantly higher (*P* = 0.0019 and *P* = 0.0020, respectively) than in the tibias with uncomplicated bone healing (2.7 ± 0.44 and 2.5 ± 0.49, respectively, *P* = 0.60 between them; Figure 5).

Furthermore, the corresponding SUV_max_ ratios were significantly higher in the osteomyelitic animals (osteomyelitis-to-intact bone ratio 1.8 ± 0.32 and 2.2 ± 0.76, respectively) than in the animals with healing bone-defects (operated-to-intact bone ratio 1.2 ± 0.18 and 1.4 ± 0.25, respectively) for both ^68^Ga-chloride and ^68^Ga-citrate (*P* = 0.012 and *P* = 0.0011, respectively) (Figure 4). In the animals with healing bone-defects, no statistically significant difference was found between the SUV_max_ ratios (*P* = 0.22, Figure 5).

### 3.3. Osteomyelitic Changes Determined Using pQCT

In pQCT imaging, the group of animals with* S. aureus* (52/52A/80) infection showed signs consistent with bone infection, characterised by cortical bone destruction with circumferential periosteal reaction, reactive endosteal new bone, and sequestrum formation. Animals with healing bone-defects showed diminution of the defect, representing cortical bone healing with no signs of infection.

### 3.4. *Ex Vivo* Analysis of ^68^Ga-Citrate Accumulation and Comparison with Previously Published ^68^Ga-Chloride Results

The* ex vivo* measurements of retrieved tissues correlated closely with the results of the* in vivo* PET imaging (Table 1). In the* ex vivo* analysis of ^68^Ga-citrate accumulation, an intense uptake was seen in osteomyelitic tibias in comparison with the contralateral intact bone (*P* < 0.0001; Table 1). In the control group, there was no statistically significant difference in ^68^Ga-citrate uptake between the operated and contralateral bones.

The accumulation of ^68^Ga-citrate was significantly (*P* < 0.0001) higher in the osteomyelitic group than in the control group of animals with healing bone-defects (Table 1). In the control group, there was no statistically significant difference in ^68^Ga-citrate uptake between the operated bone and contralateral bone.

There was no statistically significant difference in ^68^Ga-citrate accumulation in retrieved samples from blood, muscle, heart, lungs, spleen, kidney, and liver between the animals with induced osteomyelitis and animals with healing bone-defects.

Corresponding with the* in vivo* PET results, a significantly (*P* = 0.0027) higher ^68^Ga-citrate SUV-uptake was found in osteomyelitic tibias in comparison with ^68^Ga-chloride uptake. Similarly, a significant increase (*P* = 0.0026) in ^68^Ga-citrate uptake was seen in the healthy control tibias of the osteomyelitic animals in comparison with ^68^Ga-chloride uptake.

## 4. Discussion

The purpose of this study was to compare the feasibility of ^68^Ga-citrate and ^68^Ga-chloride PET/CT imaging under standardized experimental models of* S. aureus* osteomyelitis and uncomplicated bone healing. There is a clinical need for more specific and early detection of bacterial infection after bone surgery. A tracer capable of differentiating between the physiological healing processes occurring in low-grade inflammation and early bacterial infection would therefore be highly beneficial. ^18^F-FDG is a gold standard for PET imaging, but it is not specific for infection and inflammation. There are high expectations for novel tracers for the imaging of infection, with studies applying them in both patients and experimental models, with the objective of increasing the specificity of PET imaging [13, 26–30]. However, to date, none of the published novel imaging agents for infection imaging have been accepted for widespread clinical use. Only a few ^68^Ga-labelled radiopharmaceuticals are in everyday use, although their potential applications are under extensive research [13, 31–34].

Nanni and coworkers [35] published promising results on the evaluation of bone infections with ^68^Ga-citrate PET/CT. It has been shown that the image qualities of ^68^Ga (*β*^+^ decay 90%, *Eβ*^+^_max_ 2.91 MeV, *T*_1/2_ 68 minutes) and ^18^F tracers (*β*^+^ decay 97%, *Eβ*^+^_max_ 0.64 MeV, *T*_1/2_ 110 minutes) are almost equal [32, 35, 36]. ^68^Ga has a rather high positron energy and it could be expected that this would result in a lower spatial resolution in comparison with ^18^F. However, both computational analyses and experimental measurements have demonstrated that the image qualities are equal [37, 38]. Another advantage of ^68^Ga over ^18^F is the lower effective dose of ionizing radiation [38, 39].

When ^68^Ga-chloride is neutralized with 1 M sodium hydroxide, ^68^Ga is rapidly hydrolyzed. Depending on the pH and concentration, in an aqueous solution ^68^Ga occurs in a form of soluble anion called gallate, ^68^Ga(OH)_4_^−^, and/or the insoluble neutral hydroxide ^68^Ga(OH)_3_. After rapid i.v. administration to the vascular system, the radioactivity can be distributed in the blood circulation as free ^68^Ga or ^68^Ga bound to transferrin, ferritin, or lactoferrin [40]. The free ^68^Ga can be directly taken up by siderophores, which are low molecular weight chelates produced by bacteria, and which have a high affinity for gallium. The citrate is only a weak chelator* in vivo*, and after i.v. injection of ^68^Ga-citrate, ^68^Ga is rapidly released, hydrolyzed, and bound to transferrin and other plasma proteins. However, it is assumed that only a soluble gallate ^67^Ga(OH)_4_^−^ is formed* in vivo* because the citrate is able to prevent precipitation of ^67^Ga(OH)_3_ [41].

The accumulation of gallium in inflammatory or infectious sites is partly due to the increased capillary permeability associated with inflammatory reactions; gallium exits the vascular network and is trapped in the extravascular compartment [11]. As an iron analogue, it binds to circulating transferrin, and via transferrin receptors, accesses cells and evolves to a highly stable state [12]. Gallium is able to bind to activated lactoferrin in neutrophils, and to bacterial siderophores [13], and uptake in macrophages has also been demonstrated [15, 16]. Also, a direct bacterial uptake pattern of gallium has been reported [17].

In our previous study, we evaluated the uptake patterns of ^68^Ga-chloride and ^18^F-FDG in* S. aureus *osteomyelitis and uncomplicated bone healing at 2 weeks after surgery [10]. ^68^Ga-chloride was initially chosen for this further study, as a promising accumulation was seen in dynamic PET imaging when it was used as a control in a ^68^Ga-labelled oligonucleotide study [34]. Additionally, ^68^Ga-chloride was readily available at the Turku PET Centre through a relatively simple cyclotron independent manufacturing process. The accumulation of ^68^Ga-chloride in the infected bone area was high 90 minutes after intravenous injection into rats, and therefore allowed rapid infection imaging. The accumulation kinetics of ^68^Ga-chloride and ^68^Ga-citrate at the site of infected bone appeared similar and rather stable during 90–120 min (Figure 3). According to* ex vivo* gamma counting of excised tissue samples, the operated bone-to-intact bone ratio of ^68^Ga-chloride was significantly higher than that of ^68^Ga-citrate. By* in vivo* PET/CT, however, the difference between tracers was the other way around; the corresponding SUV ratio with ^68^Ga-citrate was higher. The difference between* ex vivo* and* in vivo* PET results may at least party be explained by the different accumulation times. The 20 min ^68^Ga-citrate PET acquisition started 120 min after injection while accumulation time after ^68^Ga-chloride injection was shorter, 90 min. Also,* ex vivo* analyses were performed 90 min after ^68^Ga-chloride or ^68^Ga-citrate injection.

## 5. Conclusions

Our results revealed that ^68^Ga-citrate may be superior to ^68^Ga-chloride for PET imaging of osteomyelitis in postoperative situations and further support its use for such imaging.

## Figures and Tables

**Table 1:** *Ex vivo* analysis of tracer accumulation reported as SUV.

	^68^Ga-citrate		^68^Ga-chloride [10]	
	Osteomyelitis	Bone healing	Osteomyelitis	Bone healing
Blood	1.0 ± 0.12	0.85 ± 0.41	1.4 ± 0.17	1.2 ± 0.15
Muscle, operated side	0.14 ± 0.054	0.12 ± 0.055	0.075 ± 0.032	0.055 ± 0.039
Muscle, control side	0.14 ± 0.081	0.13 ± 0.087	0.10 ± 0.057	0.071 ± 0.033
Bone, operated side	0.70 ± 0.13	0.19 ± 0.093	0.48 ± 0.19	0.28 ± 0.10
Bone, intact control	0.42 ± 0.081	0.18 ± 0.088	0.24 ± 0.044	0.31 ± 0.11
Operated bone-to-muscle	7.0 ± 4.9	1.7 ± 0.67	6.7 ± 1.2	5.6 ± 1.8
Operated bone-to-blood	0.70 ± 0.13	0.23 ± 0.033	0.36 ± 0.055	0.25 ± 0.074
Operated bone-to-intact bone	1.7 ± 0.21	1.1 ± 0.13	1.9 ± 0.56	0.92 ± 0.21

## Abbreviations

^18^F-FDG: 2-Deoxy-2-[^18^F]-fluoro-*D*-glucose
CFU: Colony-forming unit
CT: Computed tomography
MRI: Magnetic resonance imaging
PET: Positron emission tomography
pQCT: Peripheral quantitative computed tomography
ROI: Region of interest
SUV_max_: Maximum standardized uptake value
SD: Standard deviation.
